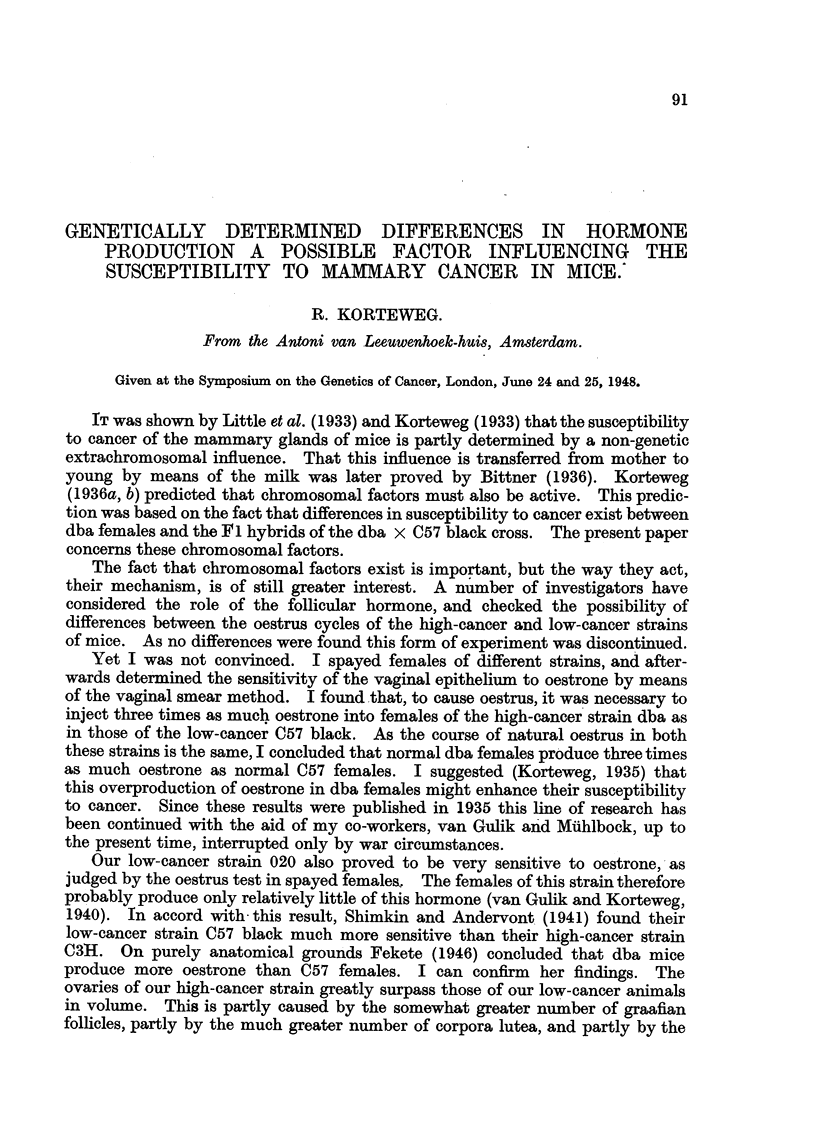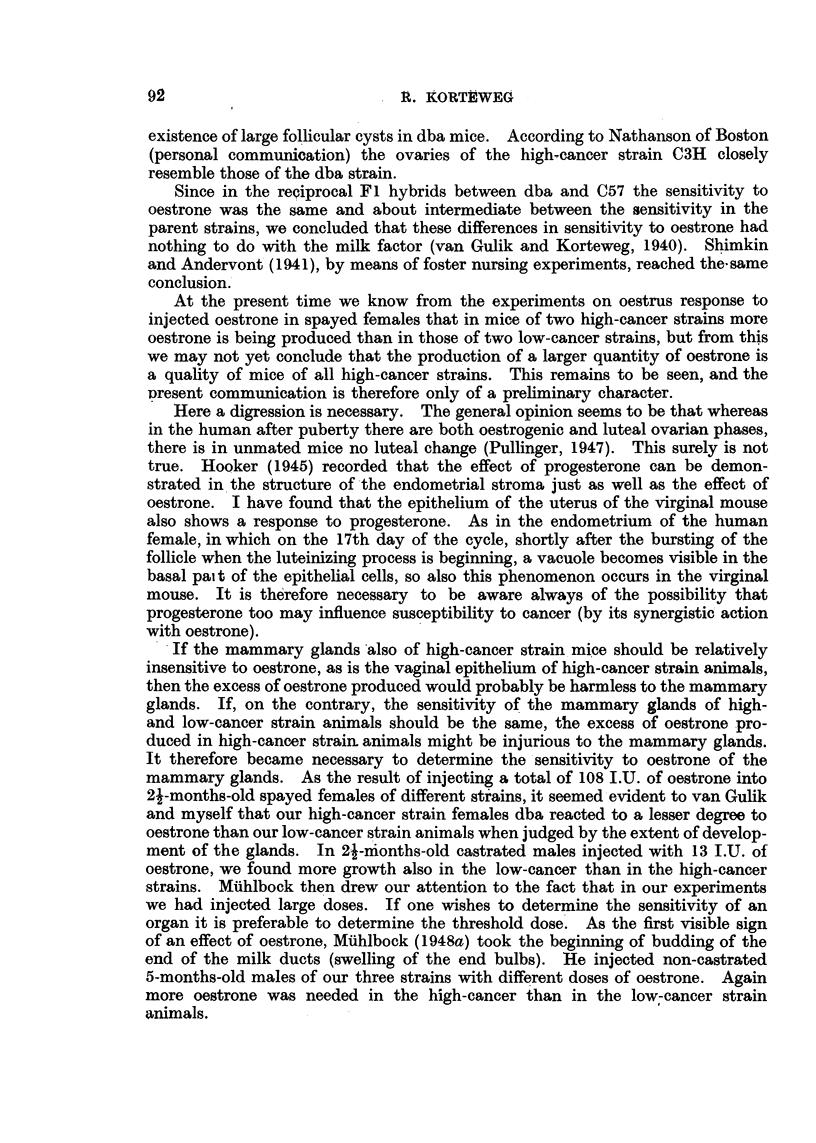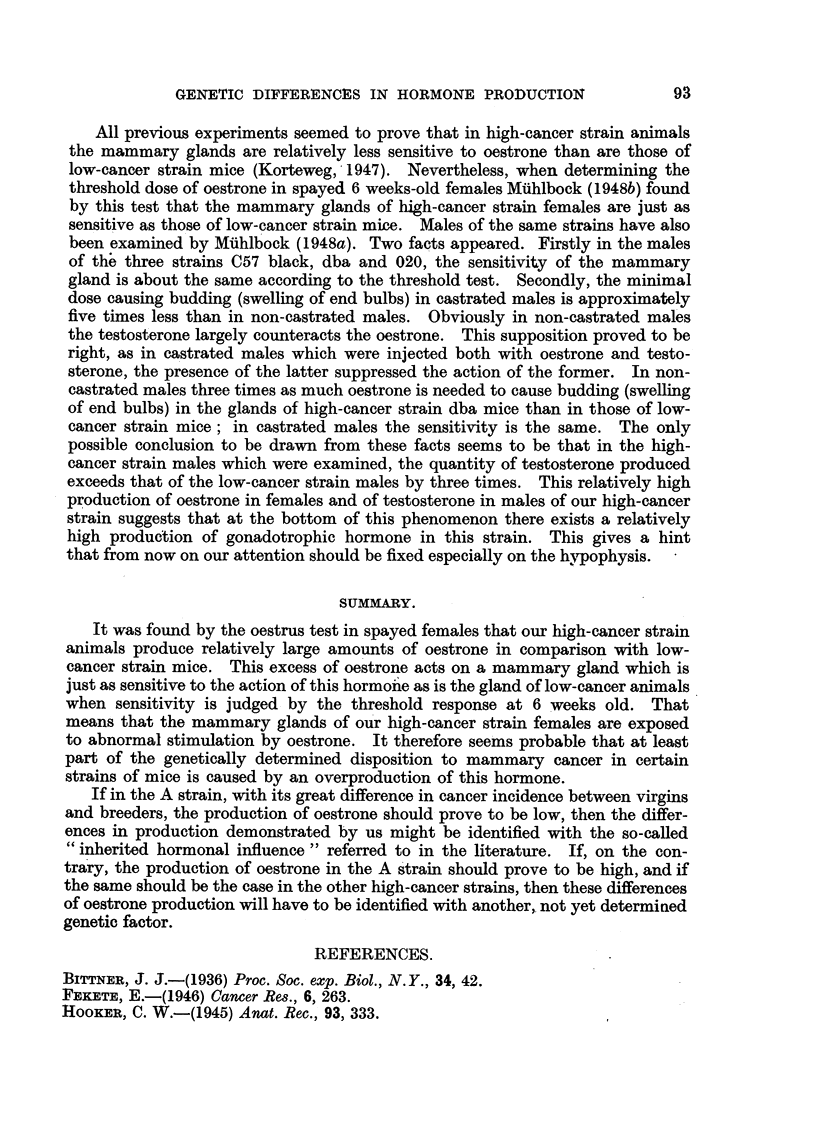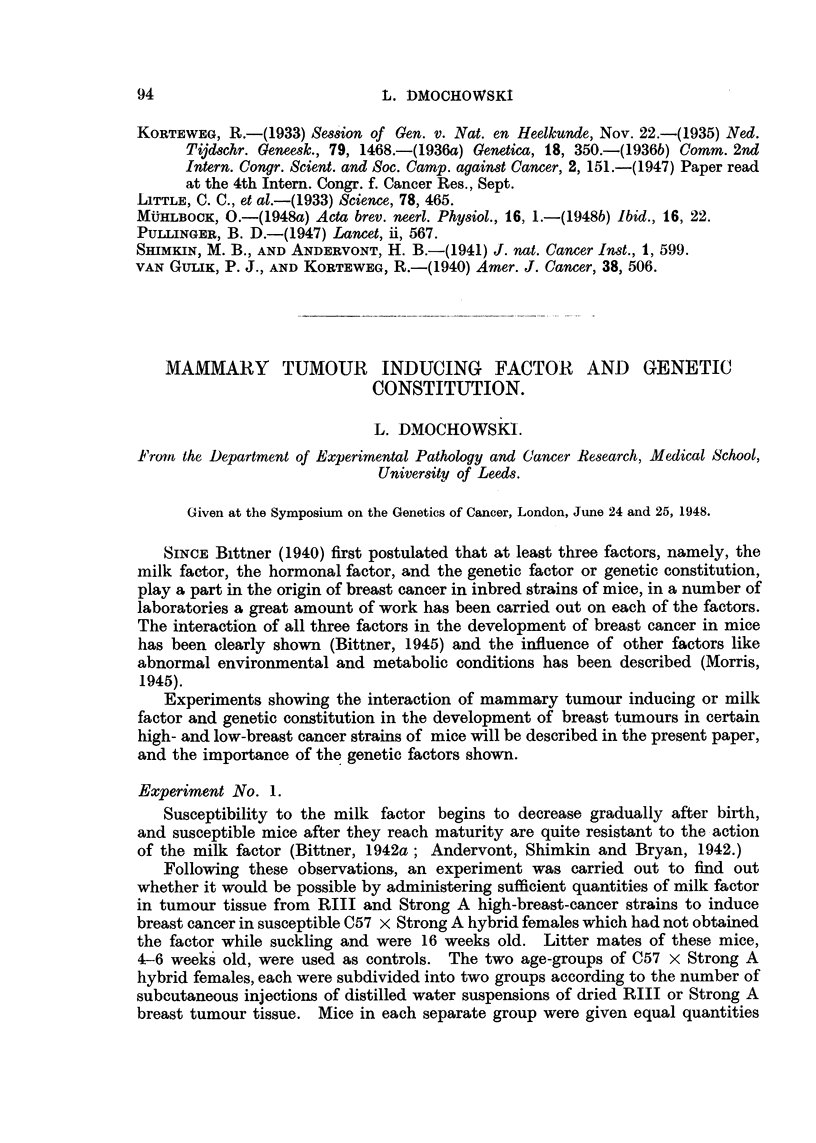# Genetically Determined Differences in Hormone Production a Possible Factor Influencing the Susceptibility to Mammary Cancer in Mice

**DOI:** 10.1038/bjc.1948.12

**Published:** 1948-06

**Authors:** R. Korteweg


### GENETICALLY DETERMINED DIFFERENCES IN HORMONE PRODUCTION A POSSIBLE FACTOR INFLUENCING THE SUSCEPTIBILITY TO MAMMARY CANCER IN MICE.

R. KORTEWEG.

*From the Antoni van Leeuwenhoek-huis, Amsterdam.*

Given at the Symposium on the Genetics of Cancer, London, June 24 and 25, 1948.

IT was shown by Little *et al.* (1933) and Korteweg (1933) that the susceptibility to cancer of the mammary glands of mice is partly determined by a non-genetic extrachromosomal influence. That this influence is transferred from mother to young by means of the milk was later proved by Bittner (1936). Korteweg (1936*a*, *b*) predicted that chromosomal factors must also be active. This prediction was based on the fact that differences in susceptibility to cancer exist between dba females and the F1 hybrids of the dba × C57 black cross. The present paper concerns these chromosomal factors.

The fact that chromosomal factors exist is important, but the way they act, their mechanism, is of still greater interest. A number of investigators have considered the role of the follicular hormone, and checked the possibility of differences between the oestrus cycles of the high-cancer and low-cancer strains of mice. As no differences were found this form of experiment was discontinued.

Yet I was not convinced. I spayed females of different strains, and afterwards determined the sensitivity of the vaginal epithelium to oestrone by means of the vaginal smear method. I found that, to cause oestrus, it was necessary to inject three times as much oestrone into females of the high-cancer strain dba as in those of the low-cancer C57 black. As the course of natural oestrus in both these strains is the same, I concluded that normal dba females produce three times as much oestrone as normal C57 females. I suggested (Korteweg, 1935) that this overproduction of oestrone in dba females might enhance their susceptibility to cancer. Since these results were published in 1935 this line of research has been continued with the aid of my co-workers, van Gulik and Mühlbock, up to the present time, interrupted only by war circumstances.

Our low-cancer strain 020 also proved to be very sensitive to oestrone, as judged by the oestrus test in spayed females. The females of this strain therefore probably produce only relatively little of this hormone (van Gulik and Korteweg, 1940). In accord with this result, Shimkin and Andervont (1941) found their low-cancer strain C57 black much more sensitive than their high-cancer strain C3H. On purely anatomical grounds Fekete (1946) concluded that dba mice produce more oestrone than C57 females. I can confirm her findings. The ovaries of our high-cancer strain greatly surpass those of our low-cancer animals in volume. This is partly caused by the somewhat greater number of graafian follicles, partly by the much greater number of corpora lutea, and partly by the existence of large follicular cysts in dba mice. According to Nathanson of Boston (personal communication) the ovaries of the high-cancer strain C3H closely resemble those of the dba strain.

Since in the reciprocal F1 hybrids between dba and C57 the sensitivity to oestrone was the same and about intermediate between the sensitivity in the parent strains, we concluded that these differences in sensitivity to oestrone had nothing to do with the milk factor (van Gulik and Korteweg, 1940). Shimkin and Andervont (1941), by means of foster nursing experiments, reached the same conclusion.

At the present time we know from the experiments on oestrus response to injected oestrone in spayed females that in mice of two high-cancer strains more oestrone is being produced than in those of two low-cancer strains, but from this we may not yet conclude that the production of a larger quantity of oestrone is a quality of mice of all high-cancer strains. This remains to be seen, and the present communication is therefore only of a preliminary character.

Here a digression is necessary. The general opinion seems to be that whereas in the human after puberty there are both oestrogenic and luteal ovarian phases, there is in unmated mice no luteal change (Pullinger, 1947). This surely is not true. Hooker (1945) recorded that the effect of progesterone can be demonstrated in the structure of the endometrial stroma just as well as the effect of oestrone. I have found that the epithelium of the uterus of the virginal mouse also shows a response to progesterone. As in the endometrium of the human female, in which on the 17th day of the cycle, shortly after the bursting of the follicle when the luteinizing process is beginning, a vacuole becomes visible in the basal part of the epithelial cells, so also this phenomenon occurs in the virginal mouse. It is therefore necessary to be aware always of the possibility that progesterone too may influence susceptibility to cancer (by its synergistic action with oestrone).

If the mammary glands also of high-cancer strain mice should be relatively insensitive to oestrone, as is the vaginal epithelium of high-cancer strain animals, then the excess of oestrone produced would probably be harmless to the mammary glands. If, on the contrary, the sensitivity of the mammary glands of high- and low-cancer strain animals should be the same, the excess of oestrone produced in high-cancer strain animals might be injurious to the mammary glands. It therefore became necessary to determine the sensitivity to oestrone of the mammary glands. As the result of injecting a total of 108 I.U. of oestrone into 2½-months-old spayed females of different strains, it seemed evident to van Gulik and myself that our high-cancer strain females dba reacted to a lesser degree to oestrone than our low-cancer strain animals when judged by the extent of development of the glands. In 2½-months-old castrated males injected with 13 I.U. of oestrone, we found more growth also in the low-cancer than in the high-cancer strains. Mühlbock then drew our attention to the fact that in our experiments we had injected large doses. If one wishes to determine the sensitivity of an organ it is preferable to determine the threshold dose. As the first visible sign of an effect of oestrone, Mühlbock (1948*a*) took the beginning of budding of the end of the milk ducts (swelling of the end bulbs). He injected non-castrated 5-months-old males of our three strains with different doses of oestrone. Again more oestrone was needed in the high-cancer than in the low-cancer strain animals.

All previous experiments seemed to prove that in high-cancer strain animals the mammary glands are relatively less sensitive to oestrone than are those of low-cancer strain mice (Korteweg, 1947). Nevertheless, when determining the threshold dose of oestrone in spayed 6 weeks-old females Mühlbock (1948*b*) found by this test that the mammary glands of high-cancer strain females are just as sensitive as those of low-cancer strain mice. Males of the same strains have also been examined by Mühlbock (1948*a*). Two facts appeared. Firstly in the males of the three strains C57 black, dba and 020, the sensitivity of the mammary gland is about the same according to the threshold test. Secondly, the minimal dose causing budding (swelling of end bulbs) in castrated males is approximately five times less than in non-castrated males. Obviously in non-castrated males the testosterone largely counteracts the oestrone. This supposition proved to be right, as in castrated males which were injected both with oestrone and testosterone, the presence of the latter suppressed the action of the former. In non-castrated males three times as much oestrone is needed to cause budding (swelling of end bulbs) in the glands of high-cancer strain dba mice than in those of low-cancer strain mice; in castrated males the sensitivity is the same. The only possible conclusion to be drawn from these facts seems to be that in the high-cancer strain males which were examined, the quantity of testosterone produced exceeds that of the low-cancer strain males by three times. This relatively high production of oestrone in females and of testosterone in males of our high-cancer strain suggests that at the bottom of this phenomenon there exists a relatively high production of gonadotrophic hormone in this strain. This gives a hint that from now on our attention should be fixed especially on the hypophysis.

### SUMMARY.

It was found by the oestrus test in spayed females that our high-cancer strain animals produce relatively large amounts of oestrone in comparison with low-cancer strain mice. This excess of oestrone acts on a mammary gland which is just as sensitive to the action of this hormone as is the gland of low-cancer animals when sensitivity is judged by the threshold response at 6 weeks old. That means that the mammary glands of our high-cancer strain females are exposed to abnormal stimulation by oestrone. It therefore seems probable that at least part of the genetically determined disposition to mammary cancer in certain strains of mice is caused by an overproduction of this hormone.

If in the A strain, with its great difference in cancer incidence between virgins and breeders, the production of oestrone should prove to be low, then the differences in production demonstrated by us might be identified with the so-called "inherited hormonal influence" referred to in the literature. If, on the contrary, the production of oestrone in the A strain should prove to be high, and if the same should be the case in the other high-cancer strains, then these differences of oestrone production will have to be identified with another, not yet determined genetic factor.

### REFERENCES.

BITTNER, J. J.—(1936) *Proc. Soc. exp. Biol., N.Y.*, **34**, 42.
FEKETE, E.—(1946) *Cancer Res.*, **6**, 263.
HOOKER, C. W.—(1945) *Anat. Rec.*, **93**, 333.

KORTEWEG, R.—(1933) *Session of Gen. v. Nat. en Heelkunde*, Nov. 22.—(1935) *Ned. Tijdschr. Geneesk.*, **79**, 1468.—(1936*a*) *Genetica*, **18**, 350.—(1936*b*) *Comm. 2nd Intern. Congr. Scient. and Soc. Camp. against Cancer*, **2**, 151.—(1947) Paper read at the 4th Intern. Congr. f. Cancer Res., Sept.
LITTLE, C. C., *et al.*—(1933) *Science*, **78**, 465.
MÜHLBOCK, O.—(1948*a*) *Acta brev. neerl. Physiol.*, **16**, 1.—(1948*b*) *Ibid.*, **16**, 22.
PULLINGER, B. D.—(1947) *Lancet*, ii, 567.
SHIMKIN, M. B., AND ANDERVONT, H. B.—(1941) *J. nat. Cancer Inst.*, **1**, 599.
VAN GULIK, P. J., AND KORTEWEG, R.—(1940) *Amer. J. Cancer*, **38**, 506.